# Contrast-Enhanced Radiologic Evaluation of Gastric Conduit Emptying After Esophagectomy

**DOI:** 10.1245/s10434-022-12596-9

**Published:** 2022-10-10

**Authors:** Minke L. Feenstra, Lily Alkemade, Janneke E. van den Bergh, Suzanne S. Gisbertz, Freek Daams, Mark I. van Berge Henegouwen, Wietse J. Eshuis

**Affiliations:** 1 Department of Surgery, Amsterdam UMC Location University of Amsterdam, Amsterdam, The Netherlands; 2 Cancer Treatment and Quality of Life, Cancer Center Amsterdam, Amsterdam, The Netherlands; 3 Amsterdam Gastroenterology Endocrinology Metabolism, Amsterdam, The Netherlands; 4 Radiology and Nuclear Medicine, Amsterdam UMC Location Vrije Universiteit Amsterdam, Amsterdam, The Netherlands; 5 Department of Surgery, Amsterdam UMC Location Vrije Universiteit Amsterdam, Amsterdam, The Netherlands

## Abstract

**Background:**

Nasogastric tube (NGT) insertion is the standard of care in many hospitals after esophagectomy for gastric conduit decompression. An upper gastrointestinal contrast passage evaluation (UGI-CE) is a diagnostic test to evaluate passage through the gastric conduit. The authors hypothesized that introducing routine UGI-CE after esophagectomy results in earlier removal of the NGT and resumption of oral intake.

**Methods:**

This retrospective study evaluated two consecutive series of patients undergoing esophagectomy, one before (control group) and one after the introduction of a routine UGI-CE on postoperative day (POD) 3 or 4 (UGI-CE group). If contrast passage was found on the UGI-CE, the NGT was capped and removed. In the control group, the NGT was routinely capped and removed on day 5 after surgery. The primary outcome was the POD on which oral diet was initiated. The secondary outcomes were the day of NGT removal, NGT reinsertions, postoperative complications, and length of hospital stay.

**Results:**

Each cohort included 74 patients. In the UGI-CE group, the contrast test was performed on median POD 3.5 (IQR, 3–4). The median day of NGT removal, initiation of clear liquids, and full liquid and solid intake was 1 to 2 days earlier in the UGI-CE group than in the control group (i.e. POD 4, 4, 5, and 6 vs. POD 5, 5, 6.5, and 8; all *p* < 0.001). The study found no significant differences in NGT reinsertions, pneumonias, anastomotic leakages, or hospital stay.

**Conclusion:**

The routine use of a UGI-CE after esophagectomy led to earlier removal of the NGT and earlier resumption of oral intake.

Esophageal cancer has the ninth highest incidence of all malignancies and is the seventh deadliest cancer worldwide.^1^ Curative treatment generally consists of neoadjuvant therapy followed by an esophagectomy. Continuity after resection of the esophagus often is restored with a gastric conduit, which is pulled up into the thoracic cavity or neck, where an esophago-gastrostomy is created.

Because esophagectomy is a technically challenging operation, postoperative morbidity generally is high, with a long hospital stay compared with other oncologic surgeries. One of the most severe complications after esophagectomy with gastric conduit reconstruction is anastomotic leakage. With an incidence of 12% for intrathoracic and 32% for cervical anastomoses in the Netherlands and an associated median hospital stay of 27 days, prevention of this complication is of great importance.^2–4^

A possible cause of anastomotic leakage is gastric distension due to delayed gastric emptying. In many centers, a nasogastric tube (NGT) is routinely placed intraoperatively to decompress the gastric conduit, avoid distension, and thus alleviate stress on the new anastomosis. Early removal of the NGT has been one of the recommendations in the Enhanced Recovery After Surgery (ERAS) guidelines to ensure a speedy recovery.^5^ However, when exactly to remove the NGT remains debatable. Some studies have even shown that the NGT can be omitted safely without significantly more adverse events.^6,7^ However, most of these studies have a small sample size, and reinsertion rates often are not reported.^7,8^ Therefore, most clinicians remain cautious regarding removal of the NGT because both distension of the gastric conduit and reinsertion of the NGT can be harmful for the healing anastomosis.

An upper gastrointestinal contrast passage evaluation (UGI-CE) with liquid contrast such as barium or ioxithalamate is a diagnostic test to evaluate passage of the contrast from the gastric conduit to the duodenum. Historically, UGI-CEs or esophagrams were performed primarily to detect anastomotic leakage.^9,10^ However, due to the low sensitivity of the UGI-CE for anastomotic leakage, other diagnostic tools, such as computed tomography scanning, have become more popular for this purpose.^11–15^ Although the UGI-CE became redundant for the detection of anastomotic leakages, it still is useful for evaluating passage from the gastric conduit to the duodenum and it may therefore show when there is no more need for an NGT in situ.

We hypothesized that a routine UGI-CE after esophagectomy results in earlier NGT removal and resumption of an oral diet without higher reinsertion or complication rates, thereby shortening hospital stay.

## Methods

### Study Design

This retrospective cohort study was conducted at the Amsterdam UMC. Ethical approval was waived by the institutional review board due to the retrospective nature of the study. Written informed consent was obtained from the patients for use of their data anonymously. Reporting of this study adhered to the STROBE guidelines.^16^

### Data Collection

This study evaluated a consecutive series of patients in 2020 undergoing esophagectomy with gastric conduit reconstruction after the introduction of a routine UGI-CE. This series was compared with a historical consecutive series of patients from June 2018 to July 2019 (control group).

The patients were selected from our prospectively maintained database. The study enrolled all patients undergoing esophagectomy with gastric conduit reconstruction. No exclusion criteria were applied. Follow-up assessment was performed up to 30 days after surgery.

### Surgical Technique for Esophagectomy

When feasible, a minimally invasive Ivor-Lewis procedure was performed. A McKeown esophagectomy was reserved for patients with proximal esophageal tumors or paratracheal lymph node metastases requiring high radiation fields. If patients were in poor general condition, a transhiatal approach was used. The surgical details are described extensively elsewhere.^17–19^

A thoracic drain was placed on the right side, and an NGT was placed intraoperatively with the tip just above the diaphragm. Aside from the esophagectomy with gastric conduit reconstruction, a feeding jejunostomy was created in all the patients using the Seldinger technique.

### Recovery Pathways With and Without Gastric-Emptying Contrast Evaluation

**Fig. 1:**
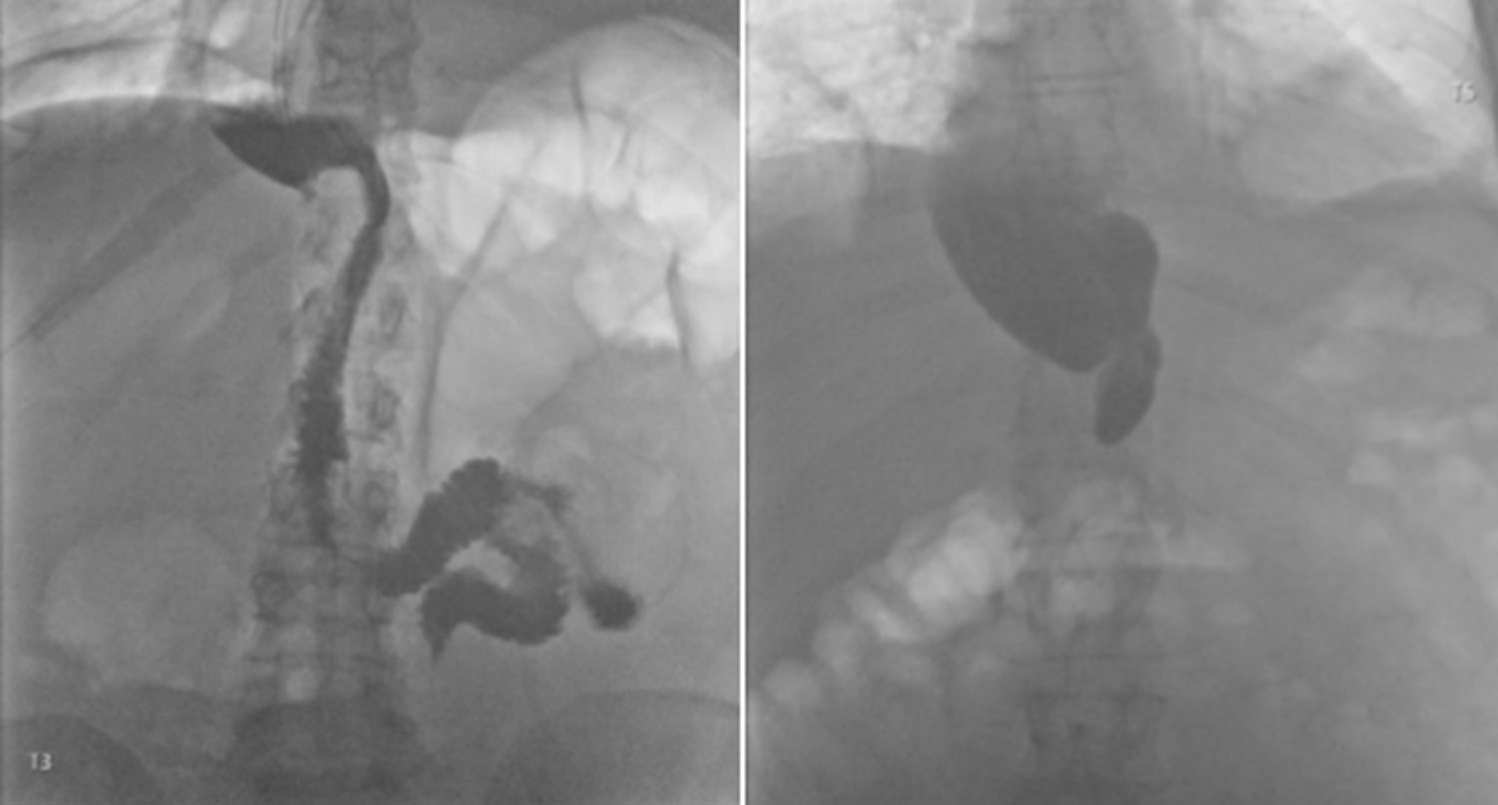
In the left image, the upper gastrointestinal contrast passage evaluation (UGI-CE) shows contrast passage through the gastric conduit. In the right image, no passage is shown

In the novel recovery pathway, the UGI-CE was conducted on POD 3 or 4 depending on the logistics in the Radiology department. The contrast agent comprised 100 ml of meglumine-ioxithalamate, or less if the patient could not drink the whole amount. For 5 min after ingestion of the contrast fluid, passage of contrast from the gastric conduit to the duodenum was evaluated with x-rays (Fig. 1). If any passage of contrast occurred within these 5 min, the NGT was capped, and if gastric retention in the following 4 h was less than 200 ml, the NGT was removed. In the pathway without a UGI-CE, the NGT was capped, checked for retention, and removed routinely on day 5 after surgery. In both pathways, after removal of the NGT, the diet was expanded from a clear liquid diet to a full liquid diet (with a higher viscosity, such as yoghurt) and finally solids.

The day after resumption of a full liquid diet, the diet was expanded to solids. The NGTs were reinserted based on clinical symptoms of gastrointestinal postoperative complications such as anastomotic leakage. The UGI-CE was not performed for detection of anastomotic leakage, but solely to evaluate contrast passage through the gastric conduit.

For all the patients, tube feeding via the jejunostomy was initiated on POD 1 (starting at 21 ml/day). Table 1 shows both recovery pathways in detail.

**Table 1:** Recovery pathways

		POD 0	POD 1	POD 2	POD 3	POD 4	POD 5	POD 6
Diagnostics	Old							Chest x-ray^a^
	New				UGI-CE^b^			
Nasogastric tube	Old	Flush 1pd 25 ml 6pd aspirate	Flush 1pd 25 ml 6pd aspirate	Flush 1pd 25 ml 6pd aspirate	Flush 1pd 25 ml 6pd aspirate	Flush 1pd 25 ml 6pd aspirate	Removed	
	New	Flush 1pd 25 ml 5pd aspirate	Flush 1pd 25 ml 5pd aspirate	Flush 1pd 25 ml 5pd aspirate	Removed^b^			
Oral diet	Old	Max 25 ml H_2_O/h	Max 25 ml H_2_O/h	Max 25 ml H_2_O/h	Max 25 ml H_2_O/h	Max 25 ml H_2_O/h or clear liquids	Clear liquids	Full liquids
	New	Max 25 ml H_2_O/h	Max 25 ml H_2_O/h	Max 25 ml H_2_O/h	Clear liquids	Full liquids	Solids	Solids
Enteral diet	Old	–	25 ml/h	45 ml/h	65 ml/h	Increase according to dietician	As POD 4	Nightly tube feeding
	New	–	21 ml/h	Increase according to dietician	Increase according to dietician	Increase according to dietician	If possible, nightly tube feeding	Nightly tube feeding
Mobilization	Old	Mobilize on the edge of the bed	> 2 h in chair or walking	> 4 h in chair or walking	> 6 h in chair or walking	> 6–8 h in chair or walking	> 8 h in chair or walking	> 8 h in chair or walking
	New	Mobilize on the edge of the bed	1x in chair, 1x walking (50 m)	2 h out of bed, 150 m walking	4,5 h out of bed, 300 m walking	6 h out of bed, 500 m walking	9 h out of bed, 1000 m walking	9 h out of bed, 1000 m walking

*POD* postoperative day; *pd* per day

^a^1 day after removal of the nasogastric tube

^b^Or POD 4

### Primary and Secondary Outcomes

The primary outcome was the first POD of resumption of an oral clear liquid diet. The secondary outcomes were the POD that a full liquid diet and solids were initiated, the POD of NGT removal, NGT reinsertion within 7 days after removal, NGT production, contrast passage through the gastric conduit on the UGI-CE, and the hospital length of stay.

Data on postoperative complications also were collected. Postoperative complications were classified according to the Clavien-Dindo classification, with Clavien-Dindo scores of 0 to 2 reflecting no or minor complications, and Clavien-Dindo scores of 3a or higher reflecting major complications.^20^

### Statistical Analysis

Data are expressed as mean ± standard deviation for normally distributed continuous variables, as median and interquartile range (IQR) for non-normally distributed continuous variables, and as proportions for binary variables. Continuous unpaired data were compared using the Mann-Whitney *U* test or the independent-samples *t* test, as appropriate.

To compare categorical data, Fisher’s exact test was used. If missing values were less than 5%, the values were not imputed, and a complete case analysis was performed. A subgroup analysis was performed for patients with no complications to only minor complications because postoperative complications can influence the outcomes regarding diet expansion and length of hospital stay.

Analyses were considered statistically significant if two-sided *p* values were lower than 0.05. All analyses were executed using SPSS version 26 (IBM Corp., Armonk, NY, USA).

## Results

From March 2020 to December 2020, 74 consecutive patients underwent esophagectomy and were prospectively included in the UGI-CE group. In a consecutive series, the same number of patients, from June 2018 to July 2019, were included in a control group.

The most commonly performed operation was a minimally invasive Ivor-Lewis procedure with a circular stapled intrathoracic anastomosis. Two patients in the UGI-CE group underwent a transhiatal esophagectomy.

The baseline characteristics of the patients are shown in Table 2. In the UGI-CE group, the UGI-CE was performed on median POD day 3.5 (IQR, 3–4). For nine patients, no UGI-CE was performed, primarily because of postoperative complications or weakness. One patient was allergic to contrast fluid and therefore did not undergo the UGI-CE. For another patient, the UGI-CE was terminated early due to gagging and suspicion of aspiration of the contrast fluid. This patient did not experience a pneumonia. Five patients were unable to drink the entire 100 ml of contrast after having no oral intake for a few days.

**Table 2:** Baseline characteristics

	UGI-CE group	Control group
	(*n* = 74)	(*n* = 74)
	*n* (%)	*n* (%)
Men	54 (73.0)	60 (81.1)
Mean age (years)	67.3 ± 9.1	63.2 ± 9.3
*ASA*		
1	2 (2.7)	2 (2.7)
2	47 (63.5)	52 (70.3)
3	25 (33.8)	20 (27.0)
Mean BMI	25.6 ± 5.2	26.3 ± 4.3
*Comorbidities*		
Cardiovascular	16 (21.6)	24 (32.4)
COPD	11 (14.9)	9 (12.2)
*Clinical T stage*		
T0	0	0
T1	6 (8.1)	2 (2.7)
T2	9 (12.2)	9 (12.2)
T3	52 (70.3)	60 (81.1)
T4	2 (2.7)	3 (4.1)
Tx	4 (5.4)	0
*Clinical N stage*		
N0	32 (43.2)	25 (33.8)
N1	22 (29.7)	34 (45.9)
N2	11 (14.9)	14 (18.9)
N3	5 (6.8)	0
Nx	3 (4.1)	1 (1.4)
*Surgical approach*		
Minimally invasive	66 (89.2)	69 (93.2)
Open	5 (6.8)	4 (5.4)
Hybrid	3 (4.1)	1 (1.4)
*Location anastomosis*		
Intrathoracic	58 (78.4)	60 (81.1)
Cervical	16 (21.6)	14 (18.9)

*UGI-CE* upper gastrointestinal contrast passage evaluation; *ASA* American Society of Anesthesiology; *BMI* body mass index; *COPD* chronic obstructive pulmonary disease

Missing data on dietary outcomes were limited (< 5%). One patient died postoperatively in the intensive care unit (ICU), not able to expand the diet before that time. One patient had anastomotic leakage with gastric conduit resection and therefore also was unable to resume an oral diet. Data on the NGT removal and reinsertions, postoperative complications, and hospital admission were complete.

### Primary and Secondary Outcomes

The median first POD of clear liquid diet initiation was 1 day earlier in the UGI-CE group than in the control group (POD 4 vs. 5; *p* < 0.001).

The median POD of resumption of full liquid and solid intake was 1 to 2 days earlier in the UGI-CE group than in the control group (namely, PODs 5 and 6 vs. 6.8 and 8, respectively, all *p* < 0.001). For 82% of the UGI-CE patients, an oral liquid diet was initiated within the first 7 postoperative days, versus 71.6% of the control patients (*p* = 0.171). The median POD of NGT removal in the UGI-CE group was POD 4, versus day 5 in the control group (*p* < 0.001). The rates of NGT reinsertion did not differ the first 7 days after removal of the NGT (Table 3).

**Table 3:** Nasogastric tube (NGT) and dietary outcomes

	UGI-CE group (*n* = 74) Median (IQR)	Control group (*n* = 74) Median (IQR)	*p* value
POD NGT removal	4 (3–5)	5 (5–6)	< 0.001
NGT reinsertion: *n* (%)	9 (12.2)	14 (18.9)	0.257
POD clear liquids	4 (3–6)	5 (5–6)	< 0.001
POD all liquids	5 (4–7)	6.5 (6–8.75)	< 0.001
POD solid intake	6 (6–8.25)	8 (7–13)	< 0.001

*UGI-CE* upper gastrointestinal contrast passage evaluation; *IQR* interquartile range; *POD* postoperative day

For seven of nine patients in the UGI-CE group with a NGT reinsertion, the UGI-CE showed contrast passage, although in three of these patients, the passage appeared to be very limited. The NGTs were reinserted due to vomiting (1 patient), a gastric acid aspiration pneumonia (1 patient), and anastomotic leakage (1 patient). In three of the four remaining patients, whose UGI-CE showed normal passage, the NGT was reinserted because of anastomotic leakage. One patient did not show passage on the UGI-CE, and after delayed removal of the NGT, the NGT was reinserted due to vomiting. One patient with an NGT reinsertion did not undergo the UGI-CE due to postoperative complications.

Four patients in the UGI-CE group showed contrast passage on the UGI-CE, but exhibited retention of more than 200 ml during 4 h after the evaluation. Therefore, their NGT was not removed the same day. The retention was measured each following day, and the NGT was removed as soon as the retention was less than 200 ml in 4 h. This resulted in removal of the NGT on POD 5 from all four patients. Three patients had the NGT removed 2 days after the UGI-CE, and one patient had the NGT removed 1 day after the UGI-CE. In two of these patients, the NGT was reinserted.

Anastomotic leakage occurred in seven patients (9.5%) in the UGI-CE group and 13 patients (17.6%) in the control group (*p* = 0.229). Pneumonia occurred in 13 patients (17.6%) and 7 patients (9.5%), respectively (*p* = 0.229). Aspiration pneumonia after the UGI-CE did not occur. The hospital length of stay did not differ between the two groups. The median POD of discharge was POD 9 in both groups (*p* = 0.529).

### Patients With No or Minor Complications

In the UGI-CE group, 58 patients (78.4%) had no complications or only minor postoperative complications (no higher than Clavien Dindo 2) versus 50 patients (67.6%) in the control group (*p* = 0.139). Table 4 shows the NGT and dietary outcomes for the patients with no complications or only minor postoperative complications, which all were similar in both groups. The length of hospital stay did not differ in the patients with no complications or minor complications between the two groups (median, 8 days; *p* = 992).

**Table 4:** Patients with no to minor postoperative complications (Clavien-Dindo 0–2)

	UGI-CE group (*n* = 58) Median (IQR)	Control group (*n* = 50) Median (IQR)	*p* value
POD NGT removal	4 (3–5)	5 (5–5)	< 0.001
NGT reinsertion: *n* (%)	4 (6.9)	4 (8.0)	0.827
POD clear liquids	4 (3–5)	5 (5–5)	< 0.001
POD all liquids	5 (4–6)	6 (6–7)	< 0.001
POD solid intake	6 (5–7)	7 (7–9)	< 0.001

*UGI-CE* upper gastrointestinal contrast passage evaluation; *IQR* interquartile range; *POD* postoperative day; *NGT* nasogastric tube

### Patients Without Passage During Gastric-Emptying Contrast Evaluation

Of 65 patients who had an UGI-CE performed, 8 did not show passage of contrast from the gastric conduit through the pylorus. If there was no passage, prokinetic agents were prescribed according to the protocol. Five patients had passage after initiation of prokinetic agents, evaluated either clinically (*n* = 3) or with another contrast UGI-CE a few days later (*n* = 2). Three patients underwent a gastric endoscopy, and for one of these patients, a pyloric balloon dilation was performed, after which there was passage through the gastric conduit. In the remaining two patients, no abnormalities were found during endoscopy. After a proton pump inhibitor was started in the one patient and a switch to another prokinetic agent was performed for the other patient, the NGTs stopped producing and were successfully removed.

The day before the UGI-CE, the median NGT production in the patients without passage was 552 ml (IQR, 394–678 ml) in 24 h versus 314 ml (IQR, 184–486 ml) in 24 h in the patients with passage through the gastric conduit (*p* = 0.030). The median overall NGT production of all the included patients was 255 ml/24 h (IQR, 120–387.5 ml/24 h) on POD 1, 346 ml/24 h (IQR, 195–510 ml/24 h) on POD 2, and 360 ml/24h (IQR, 217–604 ml/24 h) on POD 3.

## Discussion

This study evaluated the effect that the implementation of routine UGI-CE after esophagectomy has on postoperative recovery. The UGI-CE led to earlier removal of the NGT and resumption of an oral diet without an increase in NGT reinsertions or complications. There was no difference in the hospital length of stay. This also was the case for the patients with no complications or only minor complications. A significantly increased NGT production occurred for the patients without passage on the UGI-CE.

As mentioned earlier, UGI-CEs used to be performed primarily to detect anastomotic leakage.^9,10^ However, due to the low sensitivity of the contrast UGI-CE for anastomotic leakage, other diagnostic tools, such as computed tomography scanning, have become more popular for this purpose.^11–15^

To our knowledge, only Puccetti et al.^21^ previously evaluated the use of UGI-CEs for early removal of the NGT. These authors implemented a detailed postoperative protocol with the UGI-CE on postoperative day 2 or 3. Similar to the current study, they found that the UGI-CEs can be performed safely without development of contrast aspiration pneumonias. They also stated in their consecutive series that the patients undergoing UGI-CE had an earlier removal of the NGT and even a shorter hospital stay than the patients who did not undergo the evaluation. However, the patients who did not undergo the UGI-CE did not undergo the contrast evaluation due to postoperative complications. These patients were therefore already at a higher risk for a prolonged nasogastric decompression and a prolonged hospital stay. Puccetti et al.^21^ did not have a comparative group for which no standard UGI-CE was performed. Their study also included a limited number of patients.

A randomized trial by Mistry et al.^22^ evaluated early removal (on POD 2) of the NGT versus prolonged nasogastric decompression (PODs 6–10) and found early removal to be safe. The definitions of early and prolonged decompression remain arbitrary, as noted by Low,^23^ pleading for clinical and radiographic criteria for safe NGT removal. Mistry et al.^22^ also found that early NGT removal benefits patient comfort.^22^

Unfortunately, patient-reported outcome measures could not be investigated in the current study due to its retrospective design. Having the NGT in situ can cause constant aching in the throat, most intense during swallowing. Removal of the NGT is considered a relief for many patients.

This study showed that after early NGT removal, resumption of an oral diet also was initiated earlier. Multiple studies have shown that early resumption of an oral diet is just as safe as delayed resumption of an oral diet.^24–27^ Also, early NGT removal does not seem to increase the risk of anastomotic leakage, as multiple meta-analyses evaluating early removal or even omission of the NGT have found no differences in anastomotic leakage rates.^6,28^

The current study also showed no significant difference in anastomotic leakage between the groups. However, the number of anastomotic leakages was too small to provide statistical power. Although not evaluated in this study, another possible benefit of removing the NGT and resuming an oral diet early is patient comfort in the early period after discharge. Due to early resumption of an oral diet, more patients should be able to have decreased tube feeding via jejunostomy to nightly tube feeding only, and therefore to be disconnected from the feeding system during the day.

There was no difference in pneumonias between the two study groups. Contrary to the beliefs of many gastrointestinal surgeons, prolonged NGT placement did not reduce the risk of gastric acid pneumonias. The risk of pneumonia is more likely to be defined by factors other than the placement of the NGT, such as pain management and early mobilization. Some studies even suggest that an NGT in situ may increase the risk of a pneumonia due to mucus accumulation on the tube and inadequate coughing.^29,30^ Although the current study lacked statistical power for this outcome, it did not support this theory.

In the UGI-CE group, NGT production was higher in the patients without passage on the UGI-CE than in the patients with passage through the gastric conduit. Possibly, passage may be predicted by observing NGT production, allowing for more select use of the UGI-CE. The UGI-CE then could be used only when indicated. However, clinicians still must be vigilant for distension of the gastric conduit and possible anastomotic leakage.

The POD of NGT removal and resumption of an oral diet in the UGI-CE group depended not only on the UGI-CE in the early postoperative pathway, but also on capping of the NGT and measurement of the retention afterward because this was common practice in our center. Removal of the NGT can perhaps be determined by use of the UGI-CE only because the measurement of retention provides less certainty about peristalsis of the gastric conduit and absence of pylorus spasm. The measurement of retention can be influenced, for example, by oral water intake. The retention measurement also can be used before the UGI-CE. Thus, patients with little retention on POD 2 or 3 will not need a UGI-CE. The UGI-CE then can be used on a more selective basis.

Although the UGI-CE led to earlier resumption of an oral diet, it did not lead to a difference in the hospital length of stay. Possibly, the current hospital length of stay is not determined by diet resumption, but by other factors such as pain control, physical fitness, and removal of the thoracic drain. A UGI-CE still may be beneficial in an ERAS protocol because it fast-tracks the dietary outcomes. The ERAS Society recommends removal of the nasogastric tube on POD 2, when clinically appropriate.^5^ However, the ERAS Society did not describe how to evaluate whether it is clinically appropriate or not. Possibly the UGI-CE can be conducted on POD 2, thus adhering to the current guidelines but making sure that removal of the NGT is safe. The ERAS protocol by Nevo et al.^31^ even states that they are omitting the NGT as a result of a randomized trial showing that early removal is just as safe.^22^ However, early removal of the NGT and omission of the NGT are vastly different, and we recommend remaining cautious and alert for gastric distention.

To our knowledge, this was the first study to compare two series of patients, one series with and one without a UGI-CE, for early removal of the NGT.

This study had a few limitations. First, as mentioned earlier, due to the retrospective nature of this study, it was not possible to evaluate patient-reported outcomes. Second, this study evaluated not only the effect of a routinely implemented UGI-CE, but also an adjusted recovery pathway with multiple minor changes, which may have caused minor bias in this study.

In conclusion, the routine use of a UGI-CE after esophagectomy led to earlier removal of the NGT and earlier resumption of an oral diet. The implementation of the UGI-CE did not lead to more NGT reinsertions or other complications, nor did it affect the hospital length stay. More patients in the UGI-CE group were able to have only nightly tube feeding via the feeding jejunostomy at the time of discharge. The UGI-CE is a useful diagnostic test to evaluate gastric conduit passage and facilitate early NGT removal.
